# Reconfiguration of magnetic domain structures of ErFeO_3_ by intense terahertz free electron laser pulses

**DOI:** 10.1038/s41598-020-64147-5

**Published:** 2020-04-30

**Authors:** Takayuki Kurihara, Kazumasa Hirota, Hongsong Qiu, Khoa Thanh Nhat Phan, Kosaku Kato, Goro Isoyama, Makoto Nakajima

**Affiliations:** 1 Center for Applied Photonics, University of Konstanz, Universitatsstraße 10, Konstanz, Germany; 2 Institute of Laser Engineering, Osaka University, 2-6 Yamadaoka, Suita, Osaka, Japan; 3 Institute of Scientific and Industrial Research, Osaka University, 8-1 Mihogaoka, Ibaraki, Osaka, Japan, Osaka, Japan

**Keywords:** Magneto-optics, Terahertz optics, Ultrafast photonics, Condensed-matter physics, Spintronics, Free-electron lasers

## Abstract

Understanding the interaction between intense terahertz (THz) electromagnetic fields and spin systems has been gaining importance in modern spintronics research as a unique pathway to realize ultrafast macroscopic magnetization control. In this work, we used intense THz pulses with pulse energies in the order of 10 mJ/pulse generated from the terahertz free electron laser (THz-FEL) to irradiate the ferromagnetic domains of ErFeO_3_ single crystal. It was found that the domain shape can be locally reconfigured by irradiating the THz − FEL pulses near the domain boundary. Observed domain reconfiguration mechanism can be phenomenologically understood by the combination of depinning effect and the entropic force due to local thermal gradient exerted by terahertz irradiation. Our finding opens up a new possibility of realizing thermal-spin effects at THz frequency ranges by using THz-FEL pulses.

## Introduction

Application of terahertz (THz) electromagnetic field with large field amplitudes have enormously expanded in the last years as versatile means that allow for the observation of nonequilibrium electronic states in condensed matter systems, by driving their elementary degrees far beyond equilibrium^1–3^. Due to the advancement of wavelength conversion technique based on modelocked femtosecond laser sources, few-cycle THz waves with pulse energies of several microjoule order have become a common tool in ultrafast spectroscopy experiments to realize drastic changes in the macroscopic properties of correlated systems, such as conductivity^4,5^, ferroelectric polarization^6,7^, molecular orientation^8^, orbital ordering^9^ and spin orientation^10^, to count a few. Especially, the dynamics of spin systems induced by ultrastrong THz pulses have been attracting attention from the viewpoint of ultrafast spintronics technology^11–16^, wherein the final goal is to achieve macroscopic control of magnetization states in ferromagnetic domains^17–19^.

On the other hand, in optical experiments the dynamics of thermally excited spin system is gaining importance, because the photoinduced thermalization of spin systems significantly changes the equilibrium- and nonequilibrium magnetization states and allow for macroscopic change of magnetization at ultrafast time scales^20–22^. However, while there are many reports on optical heat-induced magnetic domain reversal experiments using visible and near infrared light pulses, very few works report it using THz pulses because conventionally their pulse energy are much weaker than the optical ones. From the viewpoint of high-intensity THz light sources, cutting-edge THz free electron lasers (FEL) offer pulse energies of millijoule level, which is several orders of magnitude stronger than what is available with conventional mode-locked Ti:Sapphire amplifier-based THz sources. The extremely high pulse energy contained in THz-FEL is known to enable even destructive phenomena such as desorption of molecules^23,24^ and amorphous-crystalline phase transition^5,25,26^, to count a few. In this context, one can expect that the irradiation of such ultrastrong THz-FEL pulses on spin systems could potentially lead to macroscopic change of the magnetization by strongly perturbing critical order parameters such as anisotropy, exchange interactions, and domain wall mobility^27–29^. However, little attempts have been made in such a direction so far.

In this paper, we irradiated the millijoule−level THz−FEL pulses on ferromagnetic domains of ErFeO_3_ single crystal. We found that magnetic domain shapes can be permanently reconfigured upon irradiation by THz-FEL, without causing permanent damage in the sample. The process could be explained by the combination of ultrafast heating−induced depinning effect and entropic force due to local thermal gradient. Our result demonstrates the potential of THz−FEL as a novel light source for the investigation of thermally induced spin dynamics in the THz region, and paves way for the THz spintronic devices in future.

## Methods

**Figure 1:**
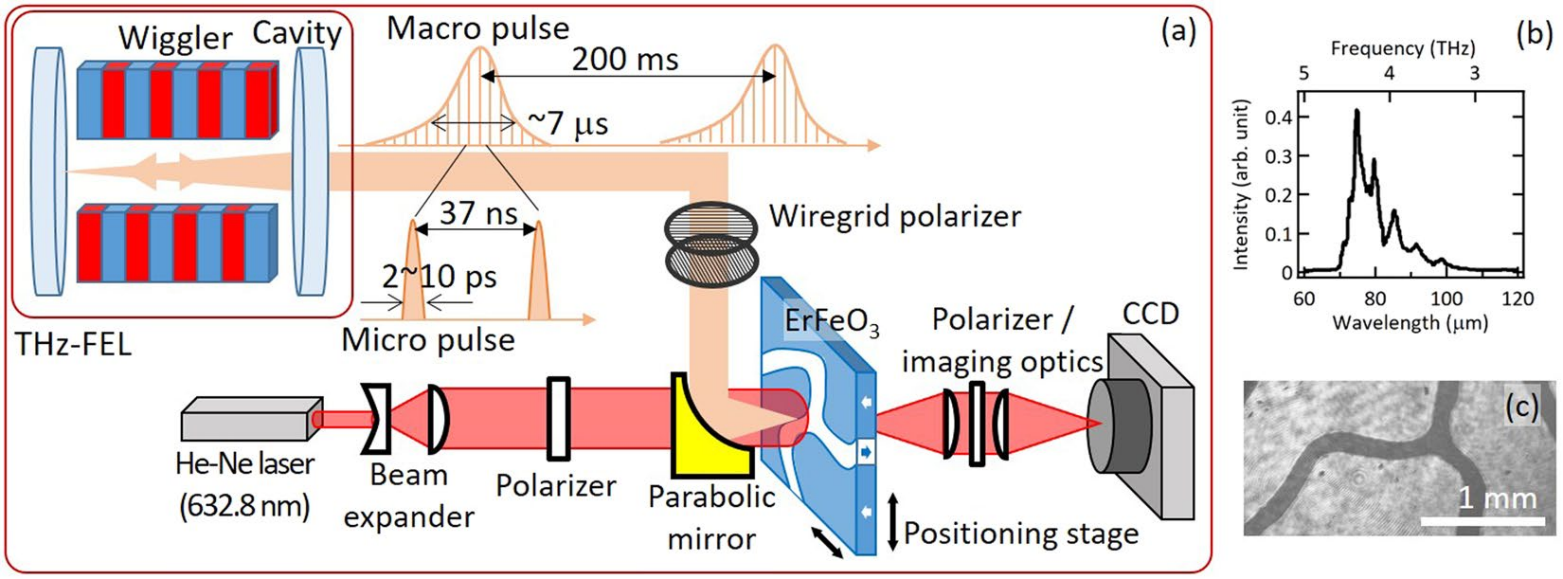
Schematic illustration of the Experimental setup. (**a**) Electron bunches injected into the wiggler placed in an optical cavity emit coherent THz pulses, which are focused on ErFeO_3_ sample. Change of the domain shape is then imaged by Faraday rotation of continuous-wave He-Ne laser with a CCD. (**b**) Spectra of the THz-FEL pulses used in the study. (**c**) Typical image of the magnetic domain structure.

The experiment was performed in the THz-FEL facility at the Institute of Scientific and Industrial Research, Osaka University, which is based on the 40 MeV L-band (1.3 GHz) electron linac. The FEL consists of a permanent magnet wiggler (a 6 cm period length and 32 periods; the magnet gap variable from 120 to 30 mm, or the K-value (rms) from 0.01 to 1.55) and a 5.531 m concentric optical cavity [Fig. 1(a)]. The linac provided an electron beam of a 15 MeV energy and approximately an 8 μs pulse duration at a repetition frequency of 5 Hz. Each pulse consists of electron bunches with approximately 4 nC charge at intervals of 37 ns for 8 μs. The repetition frequency of the electron bunches is equal to that of an optical pulse bouncing in the cavity, so that a single FEL pulse lases there. A portion of the pulse energy is extracted through a hole at the center of the upstream mirror in every round trip of the FEL pulse, so that optical pulses called micopulses are formed at intervals of 37 ns and they comprise an FEL macropulses with a duration of up to ~6 μs, which depends on operational conditions of the FEL, at the repetition frequency 5 Hz. The wavelength of the FEL beam is variable approximately from 40 to 110 μm for the electron energy of 15 MeV by varying the magnet gap of the wiggler. The maximum macropulse energy was 40 mJ or higher at a wavelength around 76 μm (4 THz) measured at the experimental station in this experiment [Fig. 1(b)]. The micropulse energy is higher than 250 μJ estimated from the maximum macropulse energy and the number of micropulses ~160, which is calculated from the duration of the macropulse divided by the micropulse intervals. The micropulse duration depends on the detuning length of the optical cavity and it is estimated to be ~2 ps at the shortest, where the macropulse energy is the highest. The peak electric field of the micropluses is estimated to be on the order of several MV/cm^24^. These pulses are focused by parabolic mirror on the *c*–cut, 100 μm−thick single crystal of ErFeO_3_. It is a typical weak ferromagnet, wherein antiferromagnetic sublattice spins are slightly canted by Dyaloshinskii−Moriya interaction and exhibits finite ferromagnetic component. At room temperature, net magnetization orients parallel to the *c* axis, i.e., magnetization is normal to the surface. Magnetic domain structure of the sample was imaged by means of Faraday rotation of the He-Ne continuous−wave laser transmitted through the sample with a CCD camera, using a pair of polarizers with their relative angles adjusted slightly off from the cross−Nicole configuration. CCD acquisition time was 0.6 s/image. Prior to the experiment we applied static magnetic field bias perpendicular to the sample, so that roughly 80% of magnetization in the sample orients towards up (down) and the rest 20% towards the opposite direction. [Fig. 1(c)]. No external magnetic field was applied on the sample during the irradiation of FEL. Focus spot diameter of the THz pulse on the surface was estimated to be approximately 200 μm (evaluated at 1/e^2^-width assuming Gaussian profile). Focus position of the FEL pulse was calibrated in advance by an additional He−Ne laser which runs parallel to the beam from FEL. Intensity and polarization of the THz pulses were adjusted by a pair of wiregrid polarizers.

## Results

**Figure 2:**
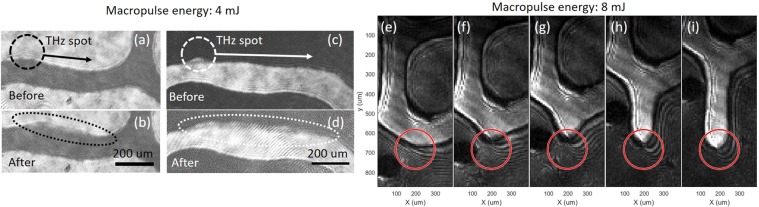
Typical images of reconfigured magnetic domain structure by FEL-irradiation. (**a–d**) Magnetic domain images before (**a,c**) and after (**b,d**) the excitation of THz-FEL with macropulse energy of approximately 4 mJ/pulse. (**e–i**) Snapshots of THz-induced domain arm extension by irradiation with macropulse energy of 8 mJ.

Figure 2(a–d) show typical Faraday rotation images of the magnetic domain observed before (a, c) and after (b, d) excitation by THz-FEL with macropulse energy of 4 mJ, measured at different initial configurations of magnetic domain structure. Black and white regions in the images represent magnetization oriented towards either +*c* or –*c* direction. Here, we first positioned THz focus spot near the flat edge of the domain arm, then scanned the focus by moving the sample stage horizontally at a speed of approximately 18 μm/s. It can be seen that after the irradiation, the edge of the minor domain regions (black (b) or white (d) parts of the image) expanded slightly from initial shape. This domain expansion can be seen more clearly when the pulse energy was increased to 8 mJ, as shown in the sequential images [Fig. 2(e–i)]. Here we focused our FEL pulses near the tip of the reconfigured domain arm and moved the spot slowly downwards. It can be clearly seen that new arm of magnetic domain was produced from pre−existing domain and can be expanded by dragging the tip of the arm downwards with the THz spot. It should be noted that the THz irradiation always resulted in the expansion of the *minor* domains, in such a way that the net magnetization of the whole sample was reduced. (i.e., when black (white) was the dominant background domain in the image, always the white (black) domain has expanded.) That means, domain structure that was generated from the previous irradiation could not be erased when we moved the THz spot back and forth across it. At the same time, when the focus spot was sufficiently far away from the domain boundaries, no local magnetization flip could be observed up to the damage threshold at macropulse energy of approximately 10 mJ. Beyond this level the sample was damaged due to the field-induced plasma generation and subsequent ablation of the surface, while below approximately 4 mJ no static change in the magnetic domain was observed. No obvious dependence on the wavelength and polarization of the incident THz pulses could be observed. The reconfigured domain shape could be easily erased by applying a weak external magnetic field (<10 mT), leaving no sign of irreversible damages on the sample.

The results obtained above point to the importance of the interaction between the existing magnetic domain boundary and the THz excitation spot. In fact, it was found that the probability of the magnetization reversal depends on the relative position of THz spot with respect to the domain boundary. To show this, in [Fig. 2(e–i)] we indicate the estimated position and focal size of the THz spot as red circles. Here, it is seen that the tip of the white arm in each image lies always in the top half part of the THz spot and not in the bottom half, seemingly following the motion of THz spot that moves away from the domain wall. Interestingly, the domain reversal point does not coincide with the center of the THz excitation spot either, where the THz field is expected to have strongest amplitude. This indicates that the domain boundary tends to move most efficiently at the trailing side of the movement that exhibits strongest spatial *gradient* of the THz power, and not the THz power itself.

**Figure 3:**
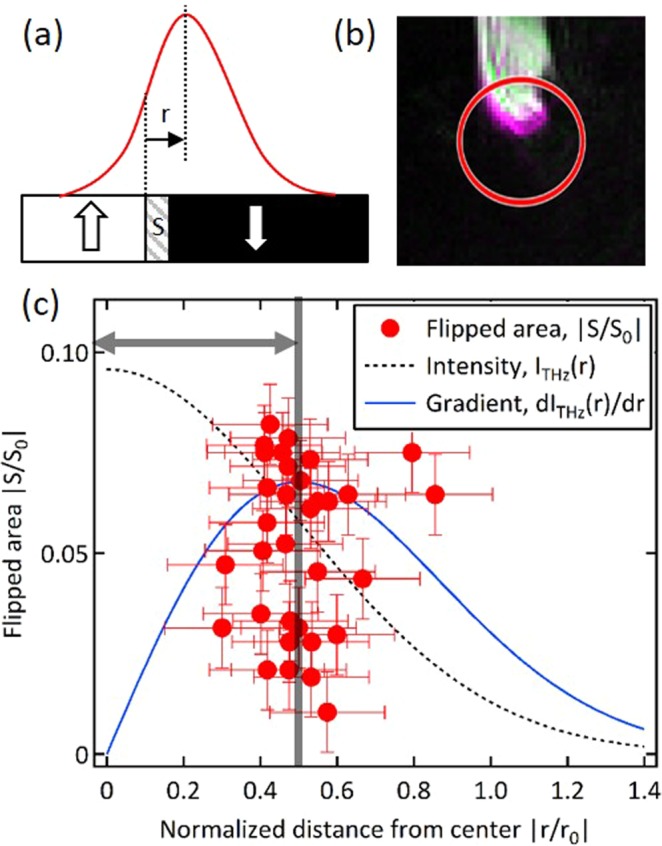
(**a**) Schematic explanation of the parameters used in the analysis. (**b**) Typical image of magnetic domain during image processing. Tip of the white domain colored by magenta indicates the region where there was reversal of magnetization from the previous frame due to THz irradiation. (**c**) Normalized flipped-domain size *S/S*_0_ plotted as a function of normalized distance *r*/*r*_0_ from the center. Here, *r*_0_ = 100 μm and *S*_0_ = π*r*_0_^2^. Black dotted- and blue curves indicate estimated Gaussian intensity profile I_THz_ (r) and its spatial derivative dI_THz_ (r)/dr, respectively. Thick black vertical line is a guide to the eye.

As further investigation, we carried out an image processing analysis by using approximately 40 sequential frames that includes [Fig. 2(e–i)]. Here, from each frame we extracted the change of the domain size *S* from the previous image caused by the THz excitation during the exposure time of CCD. At the same time, we calculate the center-of-mass position *r* of this area measured from the excitation center [Fig. 3(a,b)]. We neglected the frames which showed no or very little change of the domain shape. The result is shown in [Fig. 3(c)]. Here, *r* and *S* are normalized by the estimated spot size (*r*_0_ = 100 μm) and *S*_0_ = π*r*_0_^2^, respectively. At the same time, we also plot Gaussian function which indicates the estimated intensity profile of the THz fluence I_THz_(r) (black dotted curve). Clearly, it is seen that the magnetization flip event does not occur around the peak of THz excitation spot at the origin (*r* = 0) but rather occurs at approximately the peak of the intensity gradient profile dI_THz_(r)/dr (blue curve).

## Discussion

These results give a hint on the mechanism of the observed domain reshaping phenomena. A possible candidate is the so called entropic force^22,30–32^. It has been known that when a temperature gradient is exerted on a magnetic domain wall, there appears an effective force that acts on the wall to move it towards hotter region. This originates from the fact that in most magnetic materials, raising temperature results in the decrease of free energy of the domain wall. It is related to the increase of the domain wall entropy at high temperatures due to the reformation of magnon density of states^30,33^. In our experiment, assuming uniform excitation within the irradiated volume and taking into account the parameters used (macropulse energy: 8mJ, specific heat: 110 J/Kmol at 300 K^34^, spot radius:100 μm, sample thickness: 100 μm, coefficient of absorption at 4 THz due to the tail of optical phonon: 100 cm^−1^, molar mass: 271 g/mol and mass density: 7.86 g/cm^3^^35^), it can be estimated that the transient temperature rise in the focal spot can reach approximately 600 K, which is comparable to the Néel temperature T_N_ ~ 630 K^36^. This can result in a very strong temperature gradient in the order of ~1000 K/mm or greater. At the same time, it should also give rise to a strong softening of the magnetic domain because the magnetization and the exchange stiffness drop drastically towards T_N_. This can reduce the pininng effects of the domain wall due to impurity and defects and thus, should also increase the mobility of domain wall. Therefore, we suspect that the observed domain reshaping effect is driven by the entropic force due to THz−induced thermal gradient, assisted by the softening of exchange stiffness (depinning) due to strong heating. It should be noticed that such process can in principle also occur with the irradiation by intense visible or infrared light pulses. However, in such cases the damage induced by electronic transitions due to direct and indirect (e.g. multiphoton) absorption can take place at lower average powers. The low photon energy of the THz pulses can avoid this problem and thus enables clear observation of the abovementioned domain configuration phenomena induced by thermalization due to phonon excitation.

## Conclusion

To summarize, we observed permanent reconfiguration of the ferromagnetic domain structure in ErFeO_3_ single crystal induced by irradiation of THz−FEL with macropulse energy as high as 8 mJ. It was suggested that the domain wall motion is driven by local heating and the entropic force due to thermal gradient. Our result demonstrates the potential of THz-FEL as a novel tool to study the thermally excited spin dynamics at THz frequencies, such as ultrafast spin-Seebeck effects and magnonic spin currents in the future^37,38^.
